# New records of *Celaenorrhinus pyrrha* de Nicéville, 1889 and *C. munda* (Moore, 1884) from China (Lepidoptera, Hesperiidae)

**DOI:** 10.3897/zookeys.985.46835

**Published:** 2020-11-05

**Authors:** Gou-Xi Xue, Yutaka Inayoshi, Meng Li, Fu-Ming Zhang, Da-Kun Lai, Hai-Ying Tian

**Affiliations:** 1 School of Food and Bioengineering, Zhengzhou University of Light Industry, No. 5 Dongfeng Road, Zhengzhou, Henan, 450002, China Zhengzhou University of Light Industry Zhengzhou China; 2 Sritana Condominium 2, 96/173, Huay Kaew Rd., T. Suthep, A. Muang, Chiang Mai, 50200, Thailand Sritana Condominium Chiang Mai Thailand; 3 Forest Resource Service Center of Simian Mountain, Chongqing, 402296, China Forest Resource Service Center of Simian Mountain Chongqing China; 4 Technology Center, China Tobacco Henan Industrial Co., Ltd. No. 8 The Third Street of Zhengzhou Economic and Technological Development Zone, Zhengzhou, Henan, 450000, China China Tobacco Henan Industrial Co. Zhengzhou China

**Keywords:** COI, distribution, fauna, female genitalia, male genitalia, subspecies

## Abstract

*Celaenorrhinus pyrrha* de Nicéville, 1889, a rare species of Hesperiidae previously known to be distributed from northeastern India to Indochina, is reported from southwestern Yunnan and southwestern Chongqing, China. A 658 bp COI gene sequence of this species is published for the first time. Although Chongqing is obviously isolated from the main distribution range, morphological characters of the specimens from this locality do not indicate a subspecies differentiation. Another rare taxon, *C. munda munda* (Moore, 1884), is also recorded from China for the first time based upon a male specimen from Cuona County in the Tibet Autonomous Region. This is the second specimen of *C. munda* from China, over 100 years after the holotype of *C. munda joka* Evans, 1949. The genitalia of both species are illustrated and described. Some taxonomic notes and a distribution map are provided as well.

## Introduction

The genus *Celaenorrhinus* Hübner, [1819] includes over 100 species worldwide (Evans 1949; Yuan et al. 2015). In China, 23 species of the genus have been recorded, most of which are distributed in southern China (Wu and Hsu 2017). In this paper, two rare taxa of the genus are added to the Chinese skipper fauna, viz. *C. pyrrha* de Nicéville, 1889 and *C. munda munda* (Moore, 1884).

*Celaenorrhinus pyrrha* is known from Sikkim, Bhutan through Assam to Indochina (Evans 1949; Eliot 1959; Osada et al. 1999; Kimura et al. 2011; Ek-Amnury 2012; Nakamura and Wakahara 2012; Monastyrskii and Devyatkin 2015). In the present study, it is reported from southwestern Yunnan and southwestern Chongqing in China.

*Celaenorrhinus munda* was recorded from China by Evans (1949) as the subspecies *C. munda joka* Evans, 1949, a subspecies based on a single specimen captured from northwestern Yunnan in 1898. No additional material of this species had been found in China since. In the present study, a male specimen of *C. munda munda* is reported from southern Tibet as the easternmost record of this subspecies and the second specimen of *C. munda* from China.

Since the genitalia structures of *Celaenorrhinus pyrrha* and *C. munda* have not been illustrated in detail except for the simple hand drawings by Evans (1949: pl. 16, B.6., fig. 8; pl. 17, B.6. fig. 19), the genitalia of both taxa are illustrated and described herein. A 658 bp COI sequence of *C. pyrrha* is also provided for DNA barcoding and future molecular studies.

## Materials and methods

### Morphological examination

9♂♂, 7♀♀ of *Celaenorrhinus pyrrha* and 1♂ of *Celaenorrhinus munda munda* were studied. Specimens from China are deposited in Zhengzhou University of Light Industry, and those from Thailand and Vietnam are in the private collections of Mr. Kotaro Saito (Tokyo) and Mr. Yutaka Inayoshi (Chiang Mai). Detailed information for each specimen can be found in the Results under each species.

The terminology of morphology mainly follows those of Evans (1949), Klotz (1970) and Yuan et al. (2015).

The genitalia of both sexes were examined in glycerin and photographed using an Olympus SZX7 stereomicroscope after clearing in a cold 10% NaOH solution. Images were taken with a Canon PowerShot G16 digital camera. Image post-processing was accomplished with Adobe Photoshop CS 8.0.1.

### DNA extraction and sequence analysis

One leg of each dried specimen (Table 1) was used to extract genomic DNA following the protocol provided by DNeasy Blood and Tissue Kit (Qiagen, Germany). The partial COI gene of 658 bp was amplified by PCR using the universal primer pairs LepF and LepR, as described by Hajibabaei et al. (2006). The PCR reactions were performed in a 20 µL mixture containing 2 µL genomic DNA, 10 µL 2×Taq mix (Vazyme Biotech, China), and 0.5 µL (10 µM) forward and reverse primers. The amplification cycle was preheating at 94 °C for 3 min, then 30 cycles of 94 °C for 1 min, 50 °C for 45 sec, and 72 °C for 1 min, and a final step of 72 °C for 10 min. The PCR products were directly sequenced by Sunya Biotech, Zhengzhou, China. Multiple sequence alignments were performed in Clustal X 2.0.12 with default parameters (Thompson et al. 1997). The creditability of COI sequences was verified by BLAST and sequences were then submitted to GenBank in NCBI.

**Table 1.** Specimens used for sequencing and molecular analysis.

Species	Locality	Date	Sex	Voucher ID	Accession number
*Celaenorrhinus pyrrha*	China, Yunnan, Yingjiang, Jinzhuzhai	27.IV.2016	male	A56	MT997273
	China, Chongqing, Simian Mountain	16.VIII.2016	male	A57	MT997274
	China, Chongqing, Simian Mountain	16.VIII.2016	male	A58	MT997275
	China, Chongqing, Simian Mountain	16.VIII.2016	female	A59	MT997276
	China, Chongqing, Simian Mountain	16.VIII.2016	female	A60	MN443912
*Celaenorrhinus macrostictus*	Gabon, MDC Lonmin	29.I.2008	male	–	JN277521.1*
*Celaenorrhinus dargei*	Nigeria, Obudu Plateau	11.IV.2007	–	–	KP149680.1*
*Celaenorrhinus patula*	China, Tibet, Motuo	VII.2018	–	SCAU He1657	MN199383.1*

* Information downloaded from GenBank.

COI sequences of *Celaenorrhinus macrostictus*, *C. dargei* and *C. patula* (Table 1) were downloaded from GenBank as outgroups for the phylogenetic analysis. The software MEGA 7.0.26 (Kumar et al. 2016) was used to calculate the Kimura-2-Parameter distance and reconstruct a neighbor-joining (NJ) tree. Node support values were estimated with 1000 bootstraps replicates.

## Results

### Celaenorrhinus pyrrha

Taxon classificationAnimaliaLepidopteraHesperiidae

de Nicéville, 1889 (new record to China)

5070B2CA-4457-5988-BBB8-68D88D643B79

Figs 1–3

Celaenorrhinus pyrrha de Nicéville, 1889: 181. Type locality: Bhutan; Evans 1949: 95; Eliot 1959: 383; Osada et al. 1999: 188; Kimura et al. 2011: 33; Ek-Amnury 2012: 798; Nakamura and Wakahara 2012: 57; Monastyrskii and Devyatkin 2015: 72.

#### Material examined.

Material dissected and sequenced: 1♂ 1♀, China, Chongqing, Simian Mountain, 785 m, 16 August 2016, leg. Guoxi Xue [Dissection ID CQ164, CQ165; DNA voucher ID A58, A59]; 1♂, China, Yunnan, Yingjiang, Jinzhuzhai, 27 April 2016, leg. Guoxi Xue [Dissection ID YN25, DNA voucher ID A56]. Material dissected: 1♂ 1♀, China, Chongqing, Simian Mountain, 17 June 2007, 23 September 2007, 500–1,000 m [Dissection ID CQ125, CQ67]; 1♂ 1♀, Thailand, Nan, Doi Phu Kha, 4, 18 October 1991, leg. Inayoshi Yutaka [Dissection ID Th1, Th2]. Material sequenced: 1♂ 1♀, China, Chongqing, Simian Mountain, 785 m, 16 August 2016, leg. Guoxi Xue [DNA voucher ID A57, A60]. Other material examined: **China** • 1♀, Chongqing, Simian Mountain, 785 m, 16 August 2016, leg. Guoxi Xue; 2♀♀, Chongqing, Simian Mountain, 17 June 2007, 23 September 2007, 500–1,000 m. **Vietnam** • 1♂, Lam Dong Province, near Dalat City, Nong Trai, 1,200 m, 16 August 2003, leg. Kotaro Saito; 1♂, Dalat City, Nong Trai, 10 September 2008, leg. T. Saito; 2♂♂, Dalat City, Nong Trai, 1,200 m, 12 April 2009, 24 May 2009, leg. Kotaro Saito.

**Figures 1–4.**
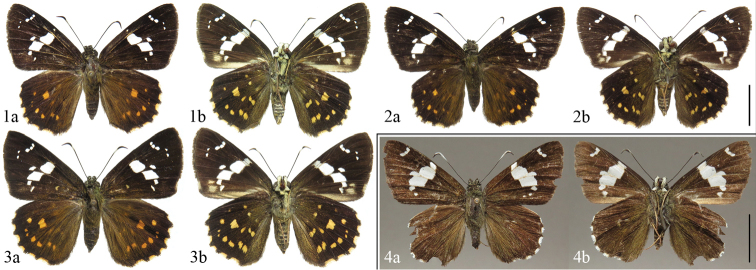
Adults of *Celaenorrhinus pyrrha* and *C. munda munda*. **1–3***C. pyrrha***1** male, China, Chongqing, Simian Mountain, 785 m, 16 August 2016, leg. Guoxi Xue [Dissection ID CQ164, DNA voucher ID A58] **2** male, China, Yunnan, Yingjiang, Jinzhuzhai, 27 April 2016, leg. Guoxi Xue [Dissection ID YN25, DNA voucher ID A56] **3** female, China, Chongqing, Simian Mountain, 785 m, 16 August 2016, leg. Guoxi Xue [DNA voucher ID A60] **4***C. munda munda*, male, China, Tibet, Cuona County, Lebugou, 19 June 2013, leg. Songyun Lang [Dissection ID Tib1] **a** dorsal side **b** ventral side. Scale bars: 1 cm.

#### Molecular analysis.

**Figure 5.**
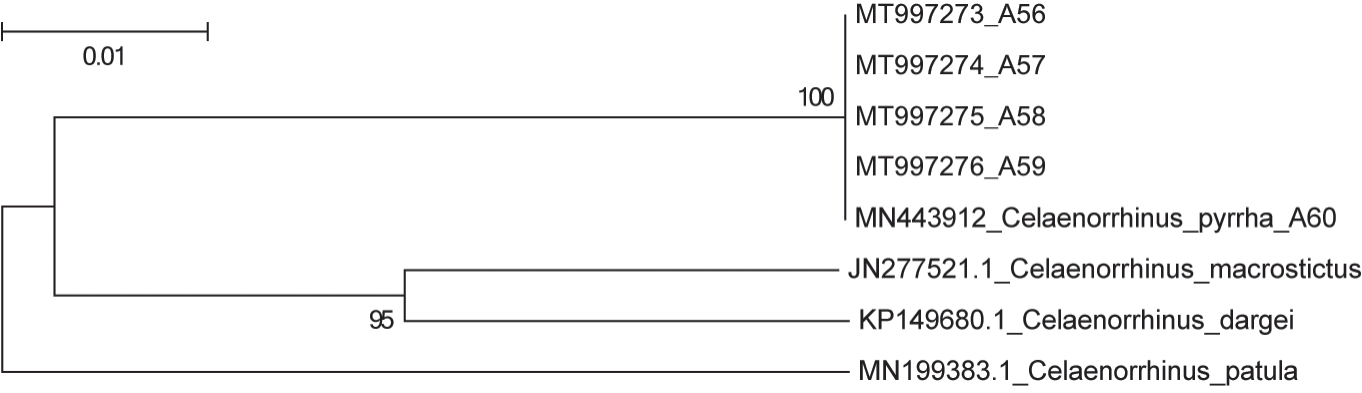
NJ tree based on Kimura-2-Parameter distances for the partial mitochondrial COI sequences of *Celaenorrhinus*, values at nodes represent the bootstrap support (BP) values.

A 658 bp partial COI sequence was successfully generated from each specimen used for DNA extraction (Table 1) via PCR and sequencing. The alignment of all the sequences used to perform a phylogenetic analysis is provided in Suppl. material 1. In the NJ tree (Fig. 5), the five voucher specimens were clustered into one clade, within which the mean K-2-P distance is 0 (Table 2), indicating they belong to the same species.

**Table 2.** Uncorrected pairwise genetic distances (Kimura-2-parameter) for the COI sequences of *Celaenorrhinus* species.

	1	2	3	4	5	6	7
1. MT997273_A56							
2. MT997274_A57	0.000						
3. MT997275_A58	0.000	0.000					
4. MT997276_A59	0.000	0.000	0.000				
5. MN443912_A60	0.000	0.000	0.000	0.000			
6. MN199383.1	0.082	0.082	0.082	0.082	0.082		
7. JN277521.1	0.075	0.075	0.075	0.075	0.075	0.089	
8. KP149680.1	0.078	0.078	0.078	0.078	0.078	0.075	0.043

#### Description.

**Figure 6.**
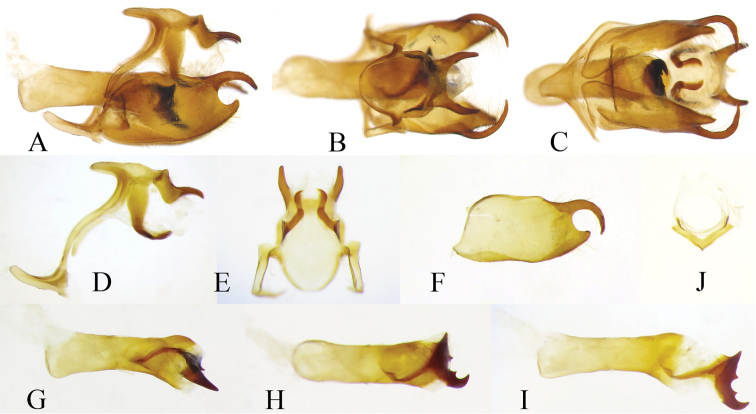
Male genitalia of *Celaenorrhinus pyrrha* from Chongqing [Dissection ID CQ125] **A** genital capsule, lateral view **B** genital capsule, dorsal view **C** genital capsule, ventral view **D** genital capsule, lateral view, valva and aedeagus removed **E** tegumen and gnathos, ventral view **F** right valva, inner surface **G** aedeagus, lateral view **I** aedeagus, with cornuti pulled out **H** aedeagus, dorsal view **J** juxta, posterior view.

**Male genitalia** (Fig. 6). In lateral view, tegumen protruding anteriorly; a small triangular plate at base of uncus; basal half of uncus pointed downwards at approximately 45°, distal half of uncus upturned and then slightly bent downwards, tapered into a sharp point; gnathos sickle-shaped, basal portion wide and elongated; saccus upturned, length about one third of the height of genitalia capsule. In dorsal view, basal half of tegumen semicircular; distal part of uncus widely bifid, tapered and bluntly pointed. In ventral view, left and right parts of gnathos separated. Distal half of valva bifid with a slender, sharply pointed dorsal branch curving downwards in lateral view and inwards in dorsal view, and a short blunt ventral branch. Aedeagus very robust; cornuti anchor shaped, extremely developed and sclerotized. Juxta V-shaped.

**Figure 7.**
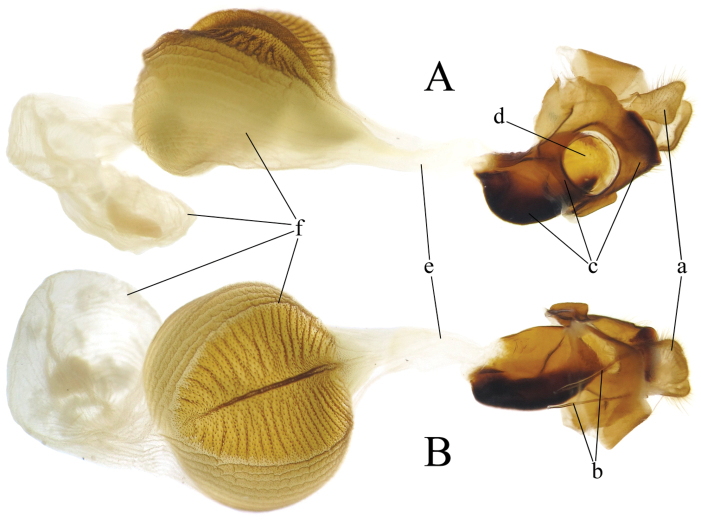
Female genitalia of *Celaenorrhinus pyrrha* from Chongqing [Dissection ID CQ67] **A** ventral view **B** dorsal view **a** papillae anales **b** apophyses posteriors **c** lamella postvaginalis-lamella antevaginalis-antrum complex **d** ostium **e** ductus bursae **f** bursa copulatrix.

**Female genitalia** (Fig. 7). Papillae anales trapezoidal, covered with short setae. Apophyses posteriors twice as long as papillae anales. Lamella postvaginalis, lamella antevaginalis and antrum merged together, with an elongated plate on each side of ostium. Ostium round, rather large. Ductus bursae short. Bursa copulatrix decorated with longitudinal striae, composed of two big bursae, dorsal side of first one coriaceous, with a longitudinal ridge and a number of transversal grooves.

#### Discussion.

**Figure 8.**
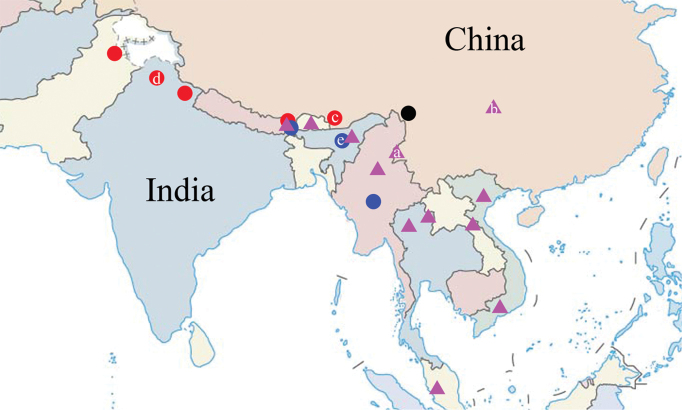
Distribution map of *Celaenorrhinus pyrrha* and *C. munda*. **Triangle***C. pyrrha***a** Yingjiang (Yunnan Province) **b** Simian Mountain (Chongqing) **Circle***C. munda***Red***C. munda munda***Blue***C. munda maculicornis***Black***C. munda joka***c** Cuona County (Tibet Autonomous Region) **d** Simla (Type locality of ssp. munda) **e** Khasi (Type locality of ssp. maculicornis).

According to our years of field surveys, and records in the literature (Evans 1949; Eliot 1959; Osada et al. 1999; Kimura et al. 2011; Ek-Amnury 2012; Monastyrskii and Devyatkin 2015), *Celaenorrhinus pyrrha* is a rather rare species throughout its distribution range (Fig. 8). In the present study, it is reported from two localities in China: Yingjiang in southwestern Yunnan, adjacent to northern Myanmar; and the Simian Mountain in southwestern Chongqing. The latter is isolated from the known distribution range of *C. pyrrha* (Evans 1949; Eliot 1959; Osada et al. 1999; Kimura et al. 2011; Ek-Amnury 2012; Monastyrskii and Devyatkin 2015) and the discovery of this species there is totally unexpected. In the present study, some minor external variations are recognized based upon examined specimens, for example: forewing length ranges from 22.4 cm to 26.0 cm; spaces C and Sc on the dorsal side of forewing may be unmarked, or with one or two small dots above the cell spot. However, we consider these as individual variations rather than intersubspecific differences because they exist in specimens from both Chongqing and Indochina. Genitalia characters of specimens from Chongqing, Yunnan, Thailand and Vietnam are compared for both sexes. According to our observations, the specimens from Chongqing cannot be distinguished by morphological characters, and thus do not represent a separate subspecies. Even so, the geographical isolation of Chongqing compared to other localities is worthy of attention, and *C. pyrrha* can possibly be expected from Guizhou, Guangxi and eastern Yunnan.

### Celaenorrhinus munda munda

Taxon classificationAnimaliaLepidopteraHesperiidae

(Moore, 1884) (new record to China)

75AC698A-D17B-5C21-BBF7-E77334425574

Fig. 4

Plesioneura munda Moore, 1884: 48, type locality: Simla, India.
Celaenorrhinus munda munda ; Evans 1949: 100.

#### Material examined.

 China • 1?, Tibet, Cuona County, Lebugou, 19 June 2013, leg. Songyun Lang.

#### Description.

**Figure 9.**
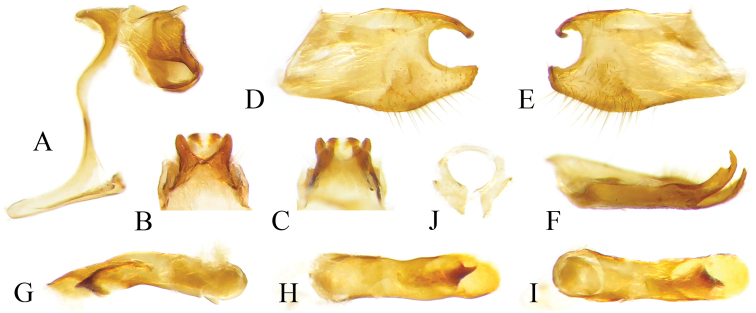
Male genitalia of *Celaenorrhinus munda munda* from Cuona, southern Tibet [Dissection ID Tib1] **A** genitalia capsule, lateral view, valva and aedeagus removed **B** tegumen and gnathos, dorsal view **C** tegumen and gnathos, ventral view **D** left valva, outer surface **E** left valva, inner surface **F** left valva, dorsal view **G** aedeagus, lateral view **H** aedeagus, dorsal view **I** aedeagus, ventral view **J** juxta, posterior view.

**Male genitalia** (Fig. 9). In lateral view, tegumen protruding anteriorly; base of uncus with a semi-erect process; basal half of uncus quadrangular, distal half narrow, sloped, sharply pointed; gnathos sickle-shaped, distal end reaching tip of uncus; saccus pointing slightly downwards, length about half the height of genitalia capsule. In dorsal view, basal processes of uncus triangular, central part of uncus constricted, distal half of uncus bifid, forming a pair of horn-shaped blunt points. In ventral view, left and right parts of gnathos separated. Distal portion of valva bifid, divided into a wide ventral branch and a slender dorsal branch, disto-dorsal margin of the latter with a notch; both branches subequal in length and bent inwards. Aedeagus robust, distal half bent downwards; cornuti triangular and sharply pointed, well sclerotized. Juxta ring-shaped.

#### Discussion.

Three subspecies have been described for *Celaenorrhinus munda*, of which *C. munda joka* Evans, 1949 is only known from the type locality, Tsekou, Yunnan, where the holotype was captured in 1898 as the only known exemplar of the species from China (Evans 1949).

*Celaenorrhinus munda maculicornis* Elwes & Edwards, 1897 is distributed from Sikkim, Assam to Myanmar (Evans 1949). The record of this subspecies from Thailand by Ek-Amnuay (2006: 752, pl. 345, H49b) was considered a misidentification of *C. leucocera* (Koller, 1844) (Ek-Amnuay et al. 2007: 14), but Ek-Amnuay (2012: 800, pl. 369, H47) included it again following Pinratana (1985: 28, 120, pl. 11, fig. 37), although Kimura et al. (2011: 34) had pointed out that Pinratana’s record is a misidentification. An earlier record of this subspecies from Thailand was listed by Godfrey (1930: 358) as *C. maculicornis*, but according to Kimura et al. (2011: 34) it is possibly a misidentification of *C. putra* (Moore). Devyatkin and Monastyrskii (1999) reported *C. munda maculicornis* from Vietnam for the first time based upon a female specimen, but in their later works, e.g., Monastyrskii and Devyatkin (2015), this name was not included, possibly because Dr. Devyatkin had realized that the specimen belongs to another species (Monastyrskii pers. comm. 2020). Therefore, we omit Vietnam from the distribution range of ssp. maculicornis (Fig. 8), since the identity of the female specimen needs further confirmation.

The nominate subspecies has been recorded from the northwestern Himalayas and Sikkim (Evans 1949). Ek-Amnuay (2006: 752, pl. 345, H49a) reported it from Thailand, but the photos of the specimens, which were provided by the second author of the present paper, actually represent *C. dhanada dhanada* (Inayoshi 2019). This mistake was corrected by Ek-Amnuay (2012: 800). Therefore, we omit Thailand from the distribution of *C. munda* in the present paper. Judging from the diagnosic characters provided by Evans (1949: 100), the specimen examined in this study belongs to *C. munda munda*. This discovery eastwardly expands the distribution range of the subspecies (Fig. 8).

Evans (1949) recorded both ssp. maculicornis and ssp. munda from Sikkim. Moreover, according to Devyatkin and Monastyrskii (1999), the two taxa are also found in Nepal, and the status of *maculicornis* remains controversial. Although differences in wing patterns of the two subspecies were clearly described by Evans (1949), the genitalia structures have not been illustrated and compared except for the simple hand drawing (Evans 1949: pl. 17, B.6. fig. 19). In future studies, comprehensive morphological and molecular analyses are needed to clarify the relationships of these taxa.

## Supplementary Material

XML Treatment for Celaenorrhinus pyrrha

XML Treatment for Celaenorrhinus munda munda

## Supplementary materials

Supplementary material 1 Alignment of sequences Data type molecular data https://binary.pensoft.net/file/471630This dataset is made available under the Open Database License (http://opendatacommons.org/licenses/odbl/1.0/). The Open Database License (ODbL) is a license agreement intended to allow users to freely share, modify, and use this Dataset while maintaining this same freedom for others, provided that the original source and author(s) are credited.Gou-Xi Xue, Yutaka Inayoshi, Meng Li, Fu-Ming Zhang, Da-Kun Lai, Hai-Ying Tian
