# Supplementary material for: New records of Celaenorrhinus pyrrha de Nicéville, 1889 and C. munda (Moore, 1884) from China (Lepidoptera, Hesperiidae)
Source: Zookeys. 2020 Nov 5;985:61–70. doi: 10.3897/zookeys.985.46835 (PMC7661477; doi:10.3897/zookeys.985.46835)
Supplement: Supplementary material 1 — Alignment of sequences [file zookeys-985-061-s001.pdf]

Supplementary file: Alignment of sequences

*Celaenorrhinus pyrrha*_A56	TACTTTATACTTTATTTTTGGAATTTGAGCAGGAATAGTA	40
*Celaenorrhinus pyrrha*_A57	--	40
*Celaenorrhinus pyrrha*_A58	--	40
*Celaenorrhinus pyrrha*_A59	--	40
*Celaenorrhinus pyrrha*_A60	--	40
Celaenorrhinus macrostictus	---------------------------------t------	40
Celaenorrhinus dargei	---------t-----------------------t------	40
Celaenorrhinus patula	---------t-----c------------------------	40

*Celaenorrhinus pyrrha*_A56	GGAACTTCTTTAAGATTATTAATTCGAACTGAATTAGGAA	80
*Celaenorrhinus pyrrha*_A57	--	80
*Celaenorrhinus pyrrha*_A58	--	80
Celaenorrhinus *pyrrha*_A59	--	80
*Celaenorrhinus pyrrha*_A60	--	80
Celaenorrhinus macrostictus	-----a---------a----------------------t-	80
Celaenorrhinus dargei	-----a---------a----------------------t-	80
Celaenorrhinus patula	------------------c-t-------------------	80

*Celaenorrhinus pyrrha*_A56	ATCCTGGATCATTAATTGGAGATGATCAAATTTATAATAC	120
*Celaenorrhinus pyrrha*_A57	--	120
*Celaenorrhinus pyrrha*_A58	--	120
*Celaenorrhinus pyrrha*_A59	--	120
*Celaenorrhinus pyrrha*_A60	--	120
Celaenorrhinus macrostictus	----a-----t-----------------------------	120
Celaenorrhinus dargei	----a-----t-----------------------------	120
Celaenorrhinus patula	----c-----tc----------------------------	120

*Celaenorrhinus pyrrha*_A56	CATTGTAACCGCTCATGCTTTTATTATAATTTTTTTTATA	160
*Celaenorrhinus pyrrha*_A57	--	160
*Celaenorrhinus pyrrha*_A58	--	160
*Celaenorrhinus pyrrha*_A59	--	160
*Celaenorrhinus pyrrha*_A60	--	160
Celaenorrhinus macrostictus	t--c-----a------------------------------	160
Celaenorrhinus dargei	t--------a-----------------------------g	160
Celaenorrhinus patula	t--------a-----------------------------g	160

*Celaenorrhinus pyrrha*_A56	GTAATACCTATTATAATTGGAGGTTTTGGAAATTGACTTG	200
*Celaenorrhinus pyrrha*_A57	--	200
*Celaenorrhinus pyrrha*_A58	--	200
*Celaenorrhinus pyrrha*_A59	--	200
*Celaenorrhinus pyrrha*_A60	--	200
Celaenorrhinus macrostictus	-----------------------a----------------	200
Celaenorrhinus dargei	-----------------------a----------------	200
Celaenorrhinus patula	--c--------------------a----------------	200

*Celaenorrhinus pyrrha*_A56	TTCCCTTAATACTAGGAGCTCCTGATATAGCTTTCCCTCG	240
*Celaenorrhinus pyrrha*_A57	--	240
*Celaenorrhinus pyrrha*_A58	--	240
*Celaenorrhinus pyrrha*_A59	--	240
*Celaenorrhinus pyrrha*_A60	--	240
Celaenorrhinus macrostictus	----t------t----------------------t-----	240
Celaenorrhinus dargei	----t------t----------------------t-----	240
Celaenorrhinus patula	----t------t-------c-----------------c--	240

*Celaenorrhinus pyrrha*_A56	AATAAATAATATAAGATTTTGATTACTGCCTCCTTCATTA	280
*Celaenorrhinus pyrrha*_A57	--	280
*Celaenorrhinus pyrrha*_A58	--	280
*Celaenorrhinus pyrrha*_A59	--	280
*Celaenorrhinus pyrrha*_A60	--	280
Celaenorrhinus macrostictus	----------------------c--t----c-----t---	280
Celaenorrhinus dargei	------c-----------------------c--c--t---	280
Celaenorrhinus patula	------------------------------c--c--c---	280

*Celaenorrhinus pyrrha*_A56	ACTCTTTTAATTTCAAGAAGAATTGTAGAAAATGGTTCAG	320
*Celaenorrhinus pyrrha*_A57	--	320
*Celaenorrhinus pyrrha*_A58	--	320
*Celaenorrhinus pyrrha*_A59	--	320
*Celaenorrhinus pyrrha*_A60	--	320
Celaenorrhinus macrostictus	--	320
Celaenorrhinus dargei	--	320
Celaenorrhinus patula	--	320

*Celaenorrhinus pyrrha*_A56	GAACAGGTTGAACAGTGTACCCCCCACTTTCAGCAAATAT	360
*Celaenorrhinus pyrrha*_A57	--	360
*Celaenorrhinus pyrrha*_A58	--	360
*Celaenorrhinus pyrrha*_A59	--	360
*Celaenorrhinus pyrrha*_A60	--	360
Celaenorrhinus macrostictus	----t-----------t--t-----t------a-c-----	360
Celaenorrhinus dargei	----t-----------t--t-----c--------t-----	360
Celaenorrhinus patula	-------------g--t-----------------t-----	360

*Celaenorrhinus pyrrha*_A56	TGCCCATCAAGGAGCTTCAGTTGATCTAGCAATTTTTTCT	400
*Celaenorrhinus pyrrha*_A57	--	400
*Celaenorrhinus pyrrha*_A58	--	400
*Celaenorrhinus pyrrha*_A59	--	400
*Celaenorrhinus pyrrha*_A60	--	400
Celaenorrhinus macrostictus	-------------------------t-------------c	400
Celaenorrhinus dargei	---t---------------------t--------------	400
Celaenorrhinus patula	------c-----------------ct----------c--c	400

*Celaenorrhinus pyrrha*_A56	TTACATTTAGCTGGAATTTCATCAATTTTAGGAGCAATTA	440
*Celaenorrhinus pyrrha*_A57	--	440
*Celaenorrhinus pyrrha*_A58	--	440
*Celaenorrhinus pyrrha*_A59	--	440
*Celaenorrhinus pyrrha*_A60	--	440
Celaenorrhinus macrostictus	-----------a--------c--t----------------	440
Celaenorrhinus dargei	-----------a--------t--t----------------	440
Celaenorrhinus patula	--------------------c--t----------------	440

*Celaenorrhinus pyrrha*_A56	ATTTTATTACAACTATTATTAATATACGAATTAGAAACTT	480
*Celaenorrhinus pyrrha*_A57	--	480
*Celaenorrhinus pyrrha*_A58	--	480
*Celaenorrhinus pyrrha*_A59	--	480
*Celaenorrhinus pyrrha*_A60	--	480
Celaenorrhinus macrostictus	-c--------t-----------------t-----------	480
Celaenorrhinus dargei	-c--------t-----------------t--c-----t--	480
Celaenorrhinus patula	-------------------------------------t--	480

*Celaenorrhinus pyrrha*_A56	ATCTTTTGATCAAATACCTTTATTTGTTTGAGCTGTAGGT	520
*Celaenorrhinus pyrrha*_A57	--	520
*Celaenorrhinus pyrrha*_A58	--	520
*Celaenorrhinus pyrrha*_A59	--	520
*Celaenorrhinus pyrrha*_A60	--	520
Celaenorrhinus macrostictus	---a---------------------a-------a-----a	520
Celaenorrhinus dargei	---a-----------------------a-----a-----a	520
Celaenorrhinus patula	------------------a--------a-----------a	520

*Celaenorrhinus pyrrha*_A56	ATTACTGCATTACTTTTATTACTCTCTTTACCTGTTCTAG	560
*Celaenorrhinus pyrrha*_A57	--	560
*Celaenorrhinus pyrrha*_A58	--	560
*Celaenorrhinus pyrrha*_A59	--	560
*Celaenorrhinus pyrrha*_A60	--	560
Celaenorrhinus macrostictus	--c--------------------t--------a--at---	560
Celaenorrhinus dargei	-----------------------t--------a--a----	560
Celaenorrhinus patula	-----a------t-ac-t-----t-----------at---	560

*Celaenorrhinus pyrrha*_A56	CAGGAGCTATCACTATATTACTAACAGATCGAAATTTAAA	600
*Celaenorrhinus pyrrha*_A57	--	600
*Celaenorrhinus pyrrha*_A58	--	600
*Celaenorrhinus pyrrha*_A59	--	600
*Celaenorrhinus pyrrha*_A60	--	600
Celaenorrhinus macrostictus	----------t---------t-------------cc-t--	600
Celaenorrhinus dargei	-t-----------c------t--------------c-t--	600
Celaenorrhinus patula	-t--------t---------t-------c--c---c-t--	600
*Celaenorrhinus pyrrha*_A56	TACATCATTTTTTGATCCAGCAGGAGGAGGAGATCCAATT	640
*Celaenorrhinus pyrrha*_A57	--	640
*Celaenorrhinus pyrrha*_A58	--	640
*Celaenorrhinus pyrrha*_A59	--	640
*Celaenorrhinus pyrrha*_A60	--	640
Celaenorrhinus macrostictus	------------------------t---------------	640
Celaenorrhinus dargei	---------c--------------t--g-----c------	640
Celaenorrhinus patula	---------c-----------------------c--t---	640
*Celaenorrhinus pyrrha*_A56	TTATATCAACATTTATTT	658
*Celaenorrhinus pyrrha*_A57	------------------	658
*Celaenorrhinus pyrrha*_A58	------------------	658
*Celaenorrhinus pyrrha*_A59	------------------	658
*Celaenorrhinus pyrrha*_A60	------------------	658
Celaenorrhinus macrostictus	-----------c------	658
Celaenorrhinus dargei	------------------	658
Celaenorrhinus patula	------------------	658
